# Specificity of cytochemical and fluorescence methods of senescence-associated β-galactosidase detection for ageing driven by replication and time

**DOI:** 10.1007/s10522-014-9505-4

**Published:** 2014-05-31

**Authors:** Patrycja Sosińska, Justyna Mikuła-Pietrasik, Monika Ryżek, Eryk Naumowicz, Krzysztof Książek

**Affiliations:** 1 Laboratory of Gerontology, Department of Pathophysiology, Poznań University of Medical Sciences, Rokietnicka 8 Str, 60-806 Poznań, Poland; 2 General Surgery Ward, Centrum Medyczne HCP, 28 Czerwca 1956 r. 223/229 Str, 61-485 Poznań, Poland

**Keywords:** Aging, Biomarker, Mesothelial cells, Replicative senescence, SA-β-Gal

## Abstract

Senescence-associated β-galactosidase (SA-β-Gal) is a widely used marker of senescent cells in vitro and in vivo. In this report, young and senescent human peritoneal mesothelial cells (HPMCs) and fragments of the omentum, from which these cells were isolated, were subjected to simultaneous examination of SA-β-Gal using two methods, i.e. cytochemical and fluorescent methods. The results obtained were confronted with the cumulative number of population doublings (CPD) and the calendar age of the tissue donor. The study showed that senescence of HPMCs proceeds with either an increased percentage of SA-β-Gal-positive cells or increased enzyme activity. Cytochemical SA-β-Gal staining in early-passage cultures negatively correlated with CPD values but not with donor age in both cell cultures and omentum specimens. Conversely, SA-β-Gal activity measured with the fluorescence method rose in proportion to the calendar age of the donor either in early-passage cultures or in primary cell isolates from omental tissue. At the same time it was not related to the CPD values. These findings may suggest that with respect to at least peritoneal mesothelial cells, the cytochemical and fluorescent methods of SA-β-Gal detection, though complementary, are informative for different levels of aging, i.e. the cytochemical approach for senescence in vitro and the fluorescence-based technique for organismal aging in vivo.

## Introduction

Replicative senescence refers to permanent growth arrest of normal somatic cells elicited by their passage through a fixed number of divisions. Until now, a network of triggers and pathways controlling cell entry into this state has been identified, thus it is tempting to think that the mechanistic aspects of senescence have already been solved (Rodier and Campisi 2011). At the same time, however, knowledge on senescence markers is rather scant. To date, various factors have been proposed as playing this role, and the most refined include the products of DNA damage (histone γ-H2A.X (von Zglinicki et al. 2005) or 8-hydroxy-2′-deoxyguanosine (Wolf et al. 2002)), tumor suppressor p16^INK4a^ (Krishnamurthy et al. 2004), and specialized domains of facultative heterochromatin (senescence-associated heterochromatin foci; SAHF) (Zhang and Adams 2007). Unfortunately, due to their primary involvement in basic cellular processes, such as cell cycle regulation or DNA damage signaling, as well as their specificity to cell type and the kind of senescence stimuli (Kosar et al. 2011), these agents do not exclusively mark senescent cells.

Despite in-depth insight into cell biology, which gave rise to knowledge pertaining to the above-mentioned molecular markers of senescence, until now the most often to be used in this context was enzyme β-galactosidase, which is detectable in the cell cytoplasm at pH 6.0. This ‘senescence-associated β-galactosidase’ (SA-β-Gal) was first reported by Dimri et al. (1995), who showed that its expression distinguishes senescent cells from proliferating and quiescent cells. Dimri et al. were also the first to show that SA-β-Gal may be useful in detecting senescent cells in vivo. Today it is well known that SA-β-Gal is of lysosomal origin and appears as the result of their increased biogenesis (Kurz et al. 2000), paralleled by increased expression of the *GLB1* gene encoding acidic lysosomal β-galactosidase (Lee et al. 2006).

Although SA-β-Gal staining has gained broad acceptance as a marker of senescence, its usage in various aspects of aging research has generated serious doubts about its specificity. Key objections include its inducibility by differentiating and oxidative agents (Frippiat et al. 2002; Untergasser et al. 2003), abundance in high-density cultures, and its absence in some tissues irrespectively of donor age (Severino et al. 2000). This criticism of SA-β-Gal combined with a lack of universality of molecular markers may imply that only the co-existence of certain biochemical, functional or molecular events may guarantee precise identification of senescent cells in vitro and in vivo. In fact, as was shown recently, confidence of senescence cell quantification could be improved by concurrent usage of a positive marker with a negative one, e.g. the presence of γ-H2A.X foci or SAHF/SA-β-Gal positivity and the absence of proliferative antigen Ki67 (Lawless et al. 2010).

In order to join this debate we designed experiments to check whether dual detection of SA-β-Gal using two different methods, namely cytochemical and fluorescent methods, could improve the reliability of this marker. Specifically, SA-β-Gal was examined in human peritoneal mesothelial cells (HPMCs), a well-established model of premature/telomere-independent cell senescence (Ksiazek 2013), and in omental tissue specimens from which those cells were isolated. Afterwards, the results obtained using both methods were confronted with a replicative cell lifespan and calendar donor age.

## Materials and methods

Unless otherwise stated, all chemicals were purchased from Sigma-Aldrich (St. Louis, MO). All tissue culture plastics were purchased from Nunc (Roskilde, Denmark).

### Cell cultures and induction of senescence

Human peritoneal mesothelial cells (HPMCs) were obtained from consenting patients undergoing abdominal surgery (Table 1). The study was approved by the institutional ethics committee. The particular protocol for cell isolation and culture conditions was described elsewhere (Ksiazek et al. 2008b). Cell senescence was induced by serial passaging at 5-day intervals until their ability to replicate was exhausted. Cultures were considered senescent when the cells developed a hypertrophic morphology and failed to increase in number for 4 weeks. Cells described as “young” were derived from the first passage. At each subcultivation the cells were counted and the cumulative number of population doublings (CPD) was estimated according to the following formula: CPD = log_2_ (C_t_/C_0_); where C_0_ indicates the number of cells at the start of incubation and C_t_ indicates the number of cells at the end of incubation.

**Table 1:** Omental tissue donor characteristics

Number	29
Gender	M/F = 12/17
Age (median and range)	45 (21–72 years)
Underlying disease	
Aortic aneurysm	6
Hernia	8
Bowel obstruction	8
Cholelithiasis	3
Acute pancreatitis	1
Colorectal cancer	3

### Detection of SA-β-Gal in vitro

For cytochemical assessment of SA-β-Gal, young and senescent HPMCs were plated in Lab-Tek™ Chamber Slides at a density of 5 × 10^4^ cells/cm^2^. After 3 days of incubation a staining protocol was performed as described by Dimri et al. (1995). For quantitative assessment of SA-β-Gal, cells were seeded in 6-well culture dishes at the same density for 3 days. Afterwards they were harvested by trypsinization, counted again and subjected to fluorescence analysis based on measurement of the rate of conversion of 4-methylumbelliferyl-β-D-galactopyranose (MUG) to 4-methylumbelliferone (4-MU), as described by Gary and Kindell (2005). The results obtained using the fluorescence method were normalized per cell autofluorescence level, which could be related to the presence and senescence-associated increase in the lipofuscin content (Ksiazek et al. 2009b).

### Detection of SA-β-Gal in vivo

Samples of the omentum were fixed in 3 % formaldehyde, embedded in paraffin, sectioned at 4 μm, deparaffinized in xylene, and rehydrated through graded alcohols to water. The mesothelium was identified using the anti-mesothelial cell antibody (clone HBME-1; Dako, Glostrup, Denmark). Cytochemical detection of SA-β-Gal was performed as described above. Planimetric analysis of the blue-stained area reflecting the presence of SA-β-Gal was conducted under a Zeiss Observer D1 microscope with AxioVision 4.6 software (Carl Zeiss, Jena, Germany). The surface of the blue staining in a given specimen was expressed as a percentage of the brown area (HBME-1-related) in a corresponding section which was treated as 100 %. In addition, a fragment of the omentum derived from the same location as the prepared sections was digested with trypsin–EDTA solution to establish a population of HPMCs that did not experience any replicative history in vitro. The isolated cells were washed with PBS and lysed, and then fluorescence assessment of SA-β-Gal was conducted.

### Statistics

Statistical analysis was performed using GraphPad Prism™ v.5.00 (GraphPad Software, San Diego, USA). The means were compared with the Wilcoxon signed-ranks test for non-parametric paired data. The correlations were analyzed using the Spearman test. Statistical significance was acknowledged when the *P* value was less than 0.05.

## Results and discussion

There is a wealth of evidence that replicative senescence of human peritoneal mesothelial cells (HPMCs) contributes to organismal, or at least omental, tissue aging. These include an age-dependent pattern of oxidative DNA damage intensification (Ksiazek et al. 2008), an accumulation of senescent cells in the omentum in vivo (Ksiazek et al. 2008b), and an inverse relationship between donor age and cell expandability in vitro (Ksiazek et al. 2007b). On the other hand, despite senescence of these cells proceeds with increased expression of numerous markers of aging, including SA-β-Gal (Książek et al. 2006), p16^INK4A^ (Ksiazek et al. 2009b), p21^CIP1^ (Ksiazek et al. 2009b), 8-OH-dG (Książek et al. 2006), lipofuscin (Ksiazek et al. 2008c), and γ-H2A.X (Ksiazek et al. 2007a), the attempts to identify a marker which would connect their senescence with organismal aging failed. In the meantime we successfully used a fluorescence-based technique for SA-β-Gal activity quantification according to the protocol described in (Gary and Kindell 2005). On this basis we collected a significant number (n = 29) of omental tissues from patients undergoing abdominal surgery, established primary cultures of HPMCs from these explants, and performed experiments aimed at answering the question of whether dual detection of SA-β-Gal with two completely different approaches, namely classic qualitative cytochemistry and quantitative fluorescence, can strengthen the enzyme’s versatility as a marker of HPMC senescence in vitro and/or omentum aging in vivo.

**Fig. 1:**
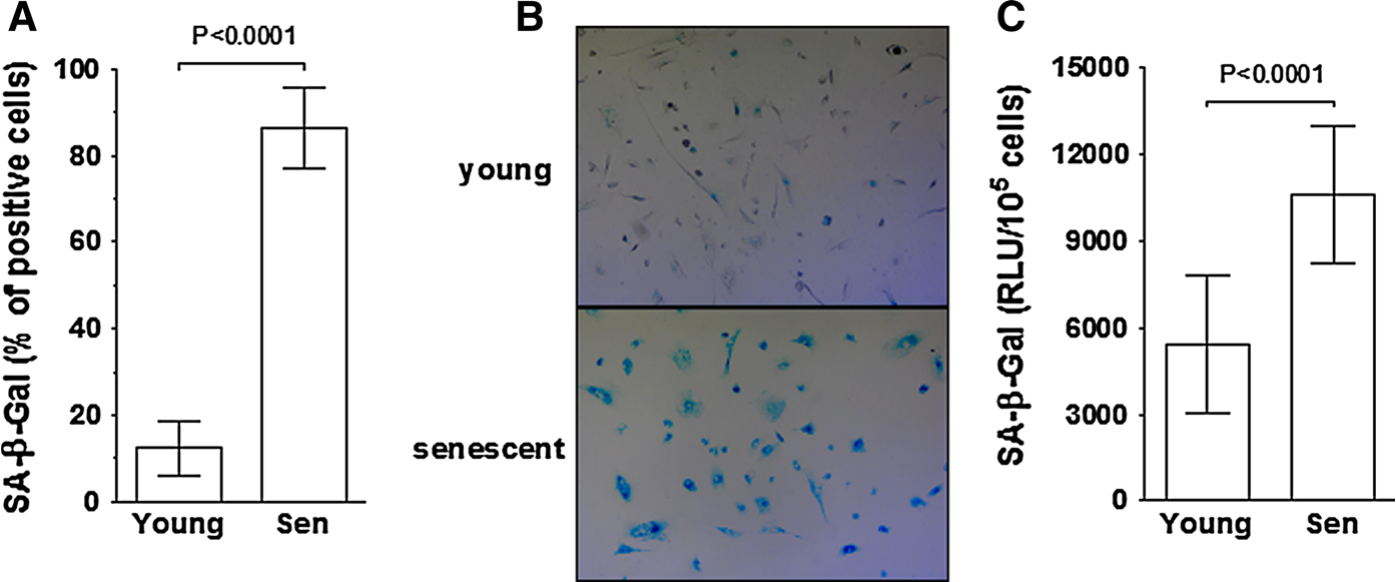
Changes in SA-β-Gal level during senescence of HPMCs. Cells were forced into senescence by serial passaging at 5-day intervals, as described in the Materials and methods. SA-β-Gal was measured in young and senescent (Sen) cells using cytochemistry (**a,b**) and fluorescence (**c**), both at pH 6.0. Results are expressed as mean ± S.D. *RLU* relative light units. Pictures in panel B show a representative result of cytochemical detection of SA-β-Gal (positive cells have the blue precipitate within the cytoplasm); magnification ×40 (**c**). Experiments were performed on cell cultures derived from 29 different donors. (Color figure online)

First of all, however, we simultaneously examined the changes in the percentage of SA-β-Gal-positive cells and the activity of the enzyme during senescence of HPMCs. This was an essential step since the latter has never been studied before. The results showed that both of the methods provide consistent information regarding the profile of SA-β-Gal changes; the cytochemistry revealed an about 7-fold increase in the size of the fraction of cells expressing SA-β-Gal (12 ± 6 vs. 87 ± 9 %; Fig. 1a) while the fluorescence showed an about 2-fold increase in enzyme activity (5433 ± 2405 vs. 10623 ± 2372 RLU/10^5^ cells, Fig. 1c). These findings are in keeping with the seminal observation of Dimri et al., who found that the level of SA-β-Gal reflects the replicative age of a culture (Dimri et al. 1995). Importantly, as we have exemplified in Fig. 1b, positivity for SA-β-Gal corresponded with hypertrophic appearance of the cells—a canonical feature of cells undergoing replicative senescence—which confirms that its appearance followed the ultimate loss of cell ability to divide and not, for instance, their response to some non-specific stimuli (Untergasser et al. 2003).

On the other hand, the results provided here, showing an explicit background level of SA-β-Gal in early-passage HPMC cultures, challenge another classic observation concerning this enzyme. Namely, sub-confluent early-passage fibroblasts and keratinocytes have been found to display only a trace level of positive cells (3 and <0.1 %, respectively) whose presence was, in fact, underestimated and treated as some kind of artifact (Dimri et al. 1995). An abundance of SA-β-Gal-positive cells in early-passage HPMC cultures was, however, indisputable and cannot be explained by inadequate culture conditions, e.g. too high culture density, as pointed out by Severino et al. (2000). In fact, HPMCs in which SA-β-Gal was determined were maintained at very low density (see the upper picture in Fig. 1b) and analyzed well before reaching their first confluency.

**Fig. 2:**
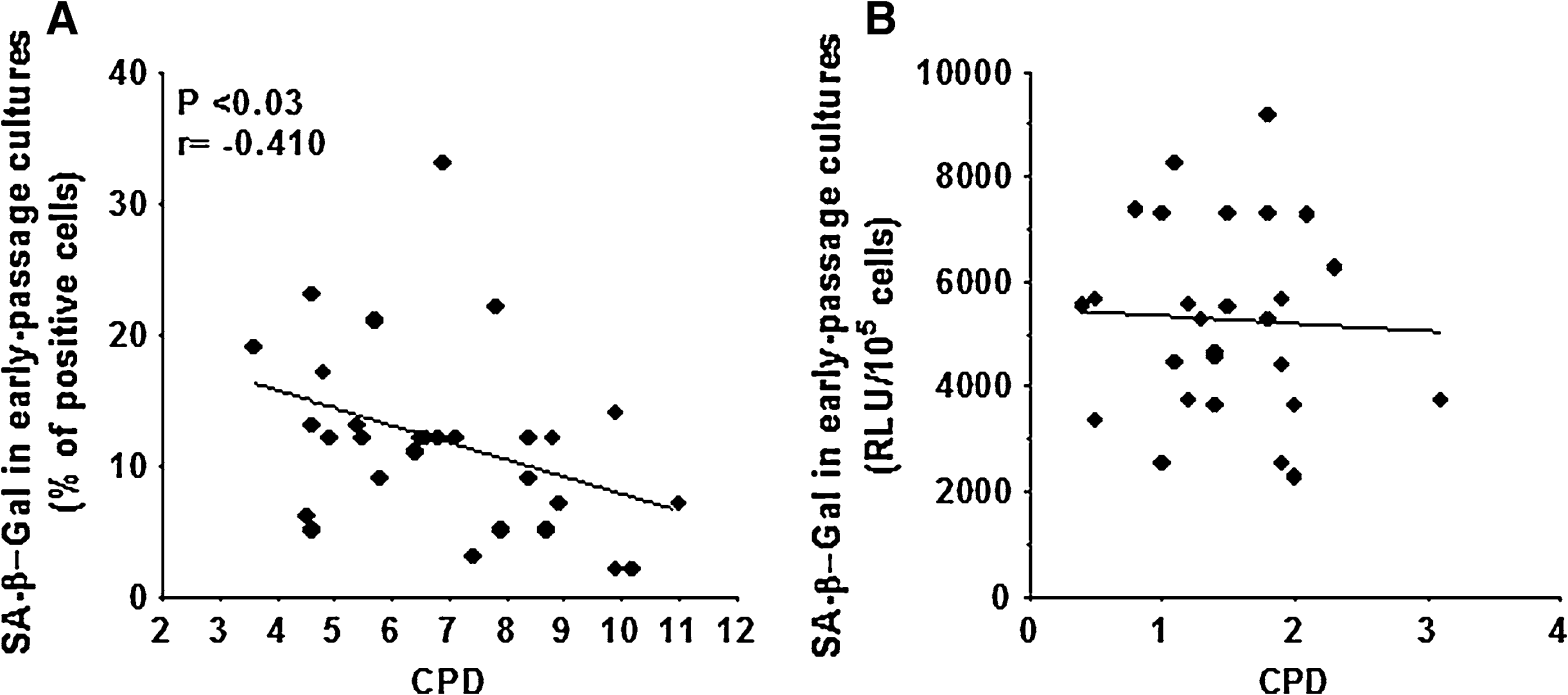
Relationship between SA-β-Gal expression (**a**) and activity (**b**) in early-passage cultures and the CPD achieved by HPMCs. Experiments were performed on cell cultures derived from 29 different donors

These findings indicate that the magnitude of SA-β-Gal in early-passage cultures is cell-specific (higher in mesothelial cells, lower in fibroblasts and keratinocytes) and that early-passage cultures are not a homogeneous population of exclusively young cells. The latter agrees with our previous studies, which showed that early-passage HPMC cultures consist of a considerable fraction of prematurely senescent cells whose presence negatively influences the replicative lifespan of a given culture (Ksiazek et al. 2008b). This scenario was also confirmed in the present study, in which the in vitro expandability of a culture, measured as the CPD achieved, inversely correlated with the percentage of SA-β-Gal-positive early-passage cells (Fig. 2a). At the same time, when the values of CPD reached by a given culture were confronted with SA-β-Gal-driven fluorescence, the relationship between these two parameters was negligible (Fig. 2b).

**Fig. 3:**
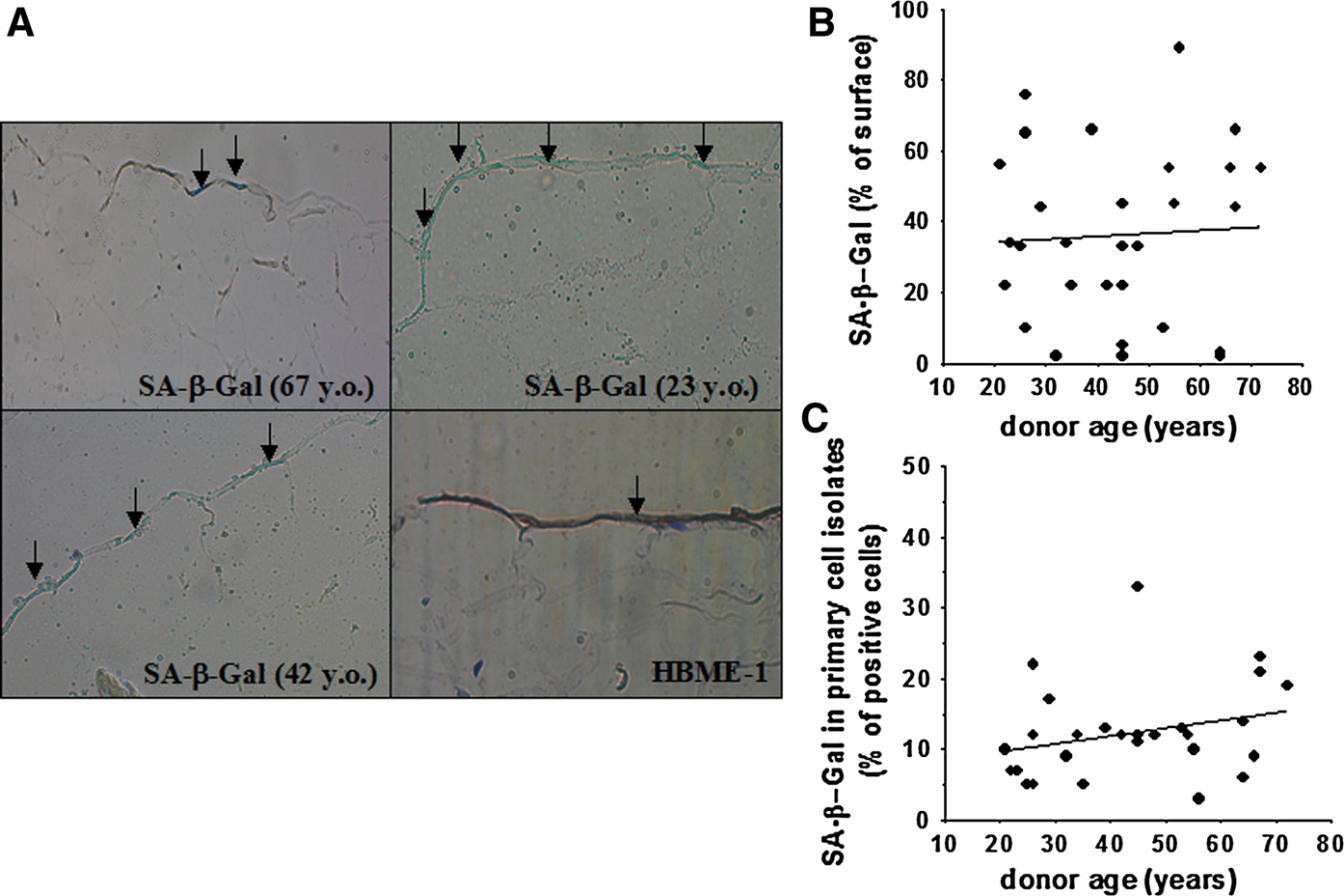
Relationship between SA-β-Gal expression in vivo and calendar age of tissue donor. Representative result of cytochemical detection of SA-β-Gal (positive cells are marked with *arrows*) in the omental tissue specimens; magnification ×40. The age of the tissue donors is shown in the brackets. The *bottom right* picture presents the results of a representative staining for the mesothelial cell-specific antigen, HBME-1 (*brown* area marked with an *arrow*) (**a**). Correlation between SA-β-Gal expression in the omental tissue and the calendar age of the tissue donor. The analysis was performed planimetrically and the blue SA-β-Gal-derived signal was expressed as a percentage (%) of the total mesothelium area delineated by HBME-1-derived *brown staining* in a corresponding section (treated as 100 %) (**b**). Correlation between SA-β-Gal expression in primary cell isolates from the omentum and the calendar age of the tissue donor (**c**). Experiments were performed on cell cultures derived from 29 different donors. (Color figure online)

After having compared both methods with respect to cell replication in vitro, we analyzed SA-β-Gal in vivo; in particular, both expression and activity of SA-β-Gal were measured in the omentum specimens as well as in primary cell isolates without any replicative history. Afterwards, the results obtained were confronted with the calendar age of the tissue donors. The study showed that the percentage of cells positive for SA-β-Gal did not correspond with organismal aging elicited by the lapse of calendar time. This was evident because the size of the fraction of cells expressing the enzyme, measured either directly in tissues (Fig. 3a, b) or in primary cell isolates (Fig. 3c), varied significantly throughout the entire spectrum of the donors’ ages. For instance, as is shown in Fig. 3a, in tissues from relatively young individuals (23 years old), 55 % of the mesothelium surface exhibited a blue SA-β-Gal-related color, whereas in explants from elder persons (67 years old) this value was as low as 2 %. These results are in conflict with a previous study on skin samples in which the authors showed that specimens from younger donors (<40 years old) did not display dermal SA-β-Gal staining, while almost all tissues from older individuals (>69 years old) did (Dimri et al., 1995). On the other hand, our findings are in line with the results of another study on skin specimens in which no age-associated increase in SA-β-Gal was found (Severino et al. 2000).

**Fig. 4:**
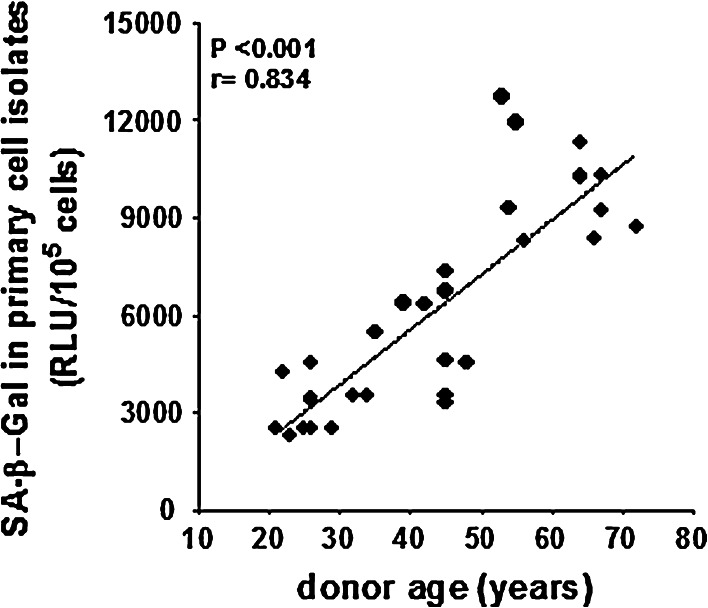
Relationship between SA-β-Gal activity in vivo and calendar age of the tissue donor. Measurements of SA-β-Gal using the fluorescence method were performed in primary cells directly released by trypsinization from the omentum. Experiments were performed on cell cultures derived from 29 different donors

At the same time, when an aliquot of primary HPMCs released by trypsinization from the omental tissue was subjected to fluorescence-based assessment of SA-β-Gal activity, a strong correlation between SA-β-Gal and the donor’s age became evident (Fig. 4). These observations may imply the possibility that frequent failures in documenting the relationship between the donor’s age and the SA-β-Gal level in tissue specimens may not be associated with the lack of such a link per se, but may rather result from the use of a non-optimal (in terms of the experimental context) technique of SA-β-Gal assessment.

It is likely that the ambiguous results of SA-β-Gal detection using both methods tested in the current study may be coupled with some of the limitations and sensitivity of a given technique. In fact, the cytochemical approach shows enzyme expression in individual cells (in some contexts this is an advantage), while fluorescence quantifies its total activity in the whole cell culture. This means that cytochemistry provides results in a binary manner (positive/negative) while fluorescence shows a full range of activities. This may lead to a situation in which the latter method, due to its higher sensitivity (Debacq-Chainiaux et al. 2009), reveals some SA-β-Gal activity level in a subset of cells that do not express the enzyme in the classic staining, e.g. due to technical purposes or the investigator’s subjectivism. These cells are, however, only pseudo-negative. An analogous scenario was described previously when the results of SA-β-Gal staining were compared with those generated by the high performance liquid chromatography (HPLC) technique (Krishna et al. 1999). In addition, one cannot exclude the possibility that the aforementioned disparities may be to some extent related to a suppression of SA-β-Gal staining in tissue sections due to some pre-analytical manipulations, such as fixation or rehydration through graded alcohols.

Linearly increasing activity of the senescence biomarker, SA-β-Gal, in the peritoneal cavity along with the patient’s age is also of special importance with respect to the intraperitoneal development of certain age-associated pathologies. One of these pathologies is ovarian malignancy, which most frequently metastasizes into the peritoneum, in particular the omentum, and whose incidence and degree of invasiveness rise exponentially with age. Because senescent HPMCs have been found to promote cancer cell adhesion (Ksiazek et al. 2009a; Ksiazek et al. 2010) and create a pro-angiogenic environment (Ksiazek et al. 2008a), their presence in the peritoneum in vivo strongly strengthens the possibility of their causative involvement in age-related exacerbation of the disease.

In conclusion, the results obtained in this report support the theory that SA-β-Gal detectable at pH 6.0 is a reliable marker of senescence/aging in vitro and in vivo. At the same time they imply an intriguing possibility—though maybe specific for the peritoneal mesothelium only—that cytochemical and fluorescent methods of SA-β-Gal assessment may be informative for replication-driven cell senescence in vitro and time-dependent organismal aging in vivo, respectively. This, in turn, indicates that although both approaches of SA-β-Gal detection seem to be complementary, the final choice of method employed should be dictated by the biological level at which aging-associated phenomena are considered.

## References

[CR1] Debacq-Chainiaux F, Erusalimsky JD, Campisi J, Toussaint O (2009). Protocols to detect senescence-associated beta-galactosidase (SA-betagal) activity, a biomarker of senescent cells in culture and in vivo. Nat Protoc.

[CR2] Dimri GP, Lee X, Basile G, Acosta M, Scott G, Roskelley C, Medrano EE, Linskens M, Rubelj I, Pereira-Smith O, Campisi J (1995). A biomarker that identifies senescent human cells in culture and in aging skin in vivo. Proc Natl Acad Sci USA.

[CR3] Frippiat C, Dewelle J, Remacle J, Toussaint O (2002). Signal transduction in H2O2-induced senescence-like phenotype in human diploid fibroblasts. Free Radic Biol Med.

[CR4] Gary RK, Kindell SM (2005). Quantitative assay of senescence-associated beta-galactosidase activity in mammalian cell extracts. Anal Biochem.

[CR5] Kosar M, Bartkova J, Hubackova S, Hodny Z, Lukas J, Bartek J (2011). Senescence-associated heterochromatin foci are dispensable for cellular senescence, occur in a cell type- and insult-dependent manner and follow expression of p16(ink4a). Cell Cycle.

[CR6] Krishna DR, Sperker B, Fritz P, Klotz U (1999). Does pH 6 beta-galactosidase activity indicate cell senescence?. Mech Ageing Dev.

[CR7] Krishnamurthy J, Torrice C, Ramsey MR, Kovalev GI, Al-Regaiey K, Su L, Sharpless NE (2004). Ink4a/Arf expression is a biomarker of aging. J Clin Invest.

[CR8] Książek K (2013). Mesothelial cell: a multifaceted model of aging. Ageing Res Rev.

[CR9] Książek K, Mikula-Pietrasik J, Catar R, Dworacki G, Winckiewicz M, Frydrychowicz M, Dragun D, Staniszewski R, Jorres A, Witowski J (2010). Oxidative stress-dependent increase in ICAM-1 expression promotes adhesion of colorectal and pancreatic cancers to the senescent peritoneal mesothelium. Int J Cancer.

[CR10] Książek K, Piwocka K, Brzezińska A, Sikora E, Zabel M, Bręborowicz A, Jorres A, Witowski J (2006). Early loss of proliferative potential of human peritoneal mesothelial cells in culture: the role of p16INK4a-mediated premature senescence. J Appl Physiol.

[CR11] Książek K, Passos JF, Olijslagers S, Saretzki G, Martin-Ruiz C, von Zglinicki T (2007). Premature senescence of mesothelial cells is associated with non-telomeric DNA damage. Biochem Biophys Res Commun.

[CR12] Książek K, Winckiewicz M, Staniszewski R, Breborowicz A, Witowski J (2007). Correlation between the donor age and the proliferative lifespan of human peritoneal mesothelial cells in vitro: is TGF-beta1 a link?. Exp Gerontol.

[CR13] Książek K, Jorres A, Witowski J (2008). Senescence induces a proangiogenic switch in human peritoneal mesothelial cells. Rejuvenation Res.

[CR14] Książek K, Mikula-Pietrasik J, Jorres A, Witowski J (2008). Oxidative stress-mediated early senescence contributes to the short replicative life span of human peritoneal mesothelial cells. Free Radic Biol Med.

[CR15] Książek K, Passos JF, Olijslagers S, von Zglinicki T (2008). Mitochondrial dysfunction is a possible cause of accelerated senescence of mesothelial cells exposed to high glucose. Biochem Biophys Res Commun.

[CR16] Książek K, Piątek K, Witowski J (2008). Impaired response to oxidative stress in senescent cells may lead to accumulation of DNA damage in mesothelial cells from aged donors. Biochem Biophys Res Commun.

[CR17] Książek K, Mikula-Pietrasik J, Korybalska K, Dworacki G, Jorres A, Witowski J (2009). Senescent peritoneal mesothelial cells promote ovarian cancer cell adhesion: the role of oxidative stress-induced fibronectin. Am J Pathol.

[CR18] Książek K, Mikula-Pietrasik J, Olijslagers S, Jorres A, von Zglinicki T, Witowski J (2009). Vulnerability to oxidative stress and different patterns of senescence in human peritoneal mesothelial cell strains. Am J Physiol Regul Integr Comp Physiol.

[CR19] Kurz DJ, Decary S, Hong Y, Erusalimsky JD (2000). Senescence-associated (beta)-galactosidase reflects an increase in lysosomal mass during replicative ageing of human endothelial cells. J Cell Sci.

[CR20] Lawless C, Wang C, Jurk D, Merz A, Zglinicki T, Passos JF (2010). Quantitative assessment of markers for cell senescence. Exp Gerontol.

[CR21] Lee BY, Han JA, Im JS, Morrone A, Johung K, Goodwin EC, Kleijer WJ, DiMaio D, Hwang ES (2006). Senescence-associated beta-galactosidase is lysosomal beta-galactosidase. Aging Cell.

[CR22] Rodier F, Campisi J (2011). Four faces of cellular senescence. J Cell Biol.

[CR23] Severino J, Allen RG, Balin S, Balin A, Cristofalo VJ (2000). Is beta-galactosidase staining a marker of senescence in vitro and in vivo?. Exp Cell Res.

[CR24] Untergasser G, Gander R, Rumpold H, Heinrich E, Plas E, Berger P (2003). TGF-beta cytokines increase senescence-associated beta-galactosidase activity in human prostate basal cells by supporting differentiation processes, but not cellular senescence. Exp Gerontol.

[CR25] von Zglinicki T, Saretzki G, Ladhoff J, d`Adda di Fagagna F, Jackson SP (2005). Human cell senescence as a DNA damage response. Mech Ageing Dev.

[CR26] Wolf FI, Torsello A, Covacci V, Fasanella S, Montanari M, Boninsegna A, Cittadini A (2002). Oxidative DNA damage as a marker of aging in WI-38 human fibroblasts. Exp Gerontol.

[CR27] Zhang R, Adams PD (2007). Heterochromatin and its relationship to cell senescence and cancer therapy. Cell Cycle.

